# Inter- and intraobserver agreement of three classification systems for lateral clavicle fractures – reliability comparison between two specialist groups

**DOI:** 10.1186/s13037-019-0228-y

**Published:** 2020-01-07

**Authors:** Thomas Rauer, Matthias Boos, Valentin Neuhaus, Prasad Ellanti, Robert Alexander Kaufmann, Hans-Christoph Pape, Florin Allemann

**Affiliations:** 1 Department of Trauma Surgery, UniversityHospital Zurich, Rämistrasse 100, Zurich, Switzerland; 2 Department of Orthopaedic Surgery, University of Pittsburgh, Pittsburgh, PA USA

**Keywords:** Lateral clavicle fracture, Reliability, Classification systems, Inter- and intraobserver agreement, Fleiss’ kappa value

## Abstract

**Background:**

Although of great value in the management of lateral clavicle fractures, substantial variation in their classification exists. We performed a retrospective study to address the inter- and intraobserver reliability of three different classification systems for lateral clavicle fractures.

**Methods:**

Radiographs of 20 lateral clavicle fractures that represented a full spectrum of adult fracture patterns were graded by five experienced radiologists and five experienced trauma surgeons according to the Orthopaedic Trauma Association (OTA), the Neer, and the Jäger/Breitner classification systems. This evaluation was performed at two different time points separated by 3 months. To measure the observer agreement, the Fleiss kappa coefficient (κ) was applied and assessed according to the grading of Landis and Koch.

**Results:**

The overall interobserver reliability showed a fair agreement in all three classification systems. For the OTA classification system, the interobserver agreement showed a mean kappa value of 0.338 ranging from 0.350 (radiologists) to 0.374 (trauma surgeons). Kappa values of the interobserver agreement for the Neer classification system ranged from 0.238 (trauma surgeons) to 0.276 (radiologists) with a mean κ of 0.278. The Jäger/Breitner classification system demonstrated a mean kappa value of 0.330 ranging from 0.306 (trauma surgeons) to 0.382 (radiologists).

The overall intraobserver reliability was moderate for the OTA and the Jäger/Breitner classification systems, while the overall intraobserver reliability for the Neer classification system was fair.

The kappa values of the intraobserver agreements showed, in all classification systems, a wide range with the OTA classification system ranging from 0.086 to 0.634, the Neer classification system ranging from 0.137 to 0.448, and a range from 0.154 to 0.625 of the Jäger/Breitner classification system.

**Conclusions:**

The low inter- and intraobserver agreement levels exhibited in all three classification systems by both specialist groups suggest that the tested lateral clavicle fracture classification systems are unreliable and, therefore, of limited value. We should recognize there is considerable inconsistency in how physicians classify lateral clavicle fractures and therefore any conclusions based on these classifications should be recognized as being somewhat subjective.

## Background

In lateral clavicle fractures, the proximity of the joint portends difficulty healing and may compromise long-term outcomes. Both operative and nonoperative management options have been advocated and remain without consensus opinion [1–8]. Various classification systems have been established for the description of clavicle fractures [9–12] and, yet, only the Neer, the modified Neer, and a new classification system described by Cho et al. have had their reliability assessed [13–15]. In general, classification systems should accurately identify injury patterns to determine prognoses, to guide treatment decisions and have to be both reliable and valid [16]. In order to be classified as a valid classification system, reliability is crucial [16]. Validity is defined as the accuracy with which the classification system describes the true pathologic process and reliability is defined as the precision of a classification system [16]. We have to distinguish between interobserver reliability, the agreement between different observers, and the intraobserver reliability, the agreement of one observer’s repeated classifications of an entity [16]. The aim of this study was to verify the reliability of three commonly used classification systems for lateral clavicle fractures by evaluating the inter- and the intraobserver agreement among two specialist groups.

## Methods

### Study design

This study was approved by the institutional review board (Business Administration System for Ethics Committees, BASEC, No. 2018–00146).

Standardized X-ray images from a total of 20 patients with a lateral clavicle fracture at a single academic level 1 trauma center were scanned and uploaded using a web-based survey system. Ten independent investigators that were employed at a level 1 trauma center and included five consultants from the Department of Radiology and five consultants from the Department of Traumatology, were invited and completed the survey. The radiologists and traumatologists averaged 5.4 years (range: 4–7 years) and 10.2 years (range: 5–17 years) of postresidency experience, respectively. The investigators were not involved in the treatment of these patients, did not receive any remuneration for their efforts and are not co-authors of this study.

**Fig. 1:**
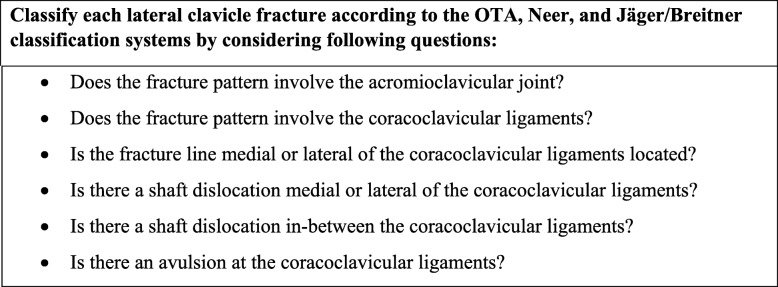
Investigator’s questionnaire for fracture classification

**Fig. 2:**
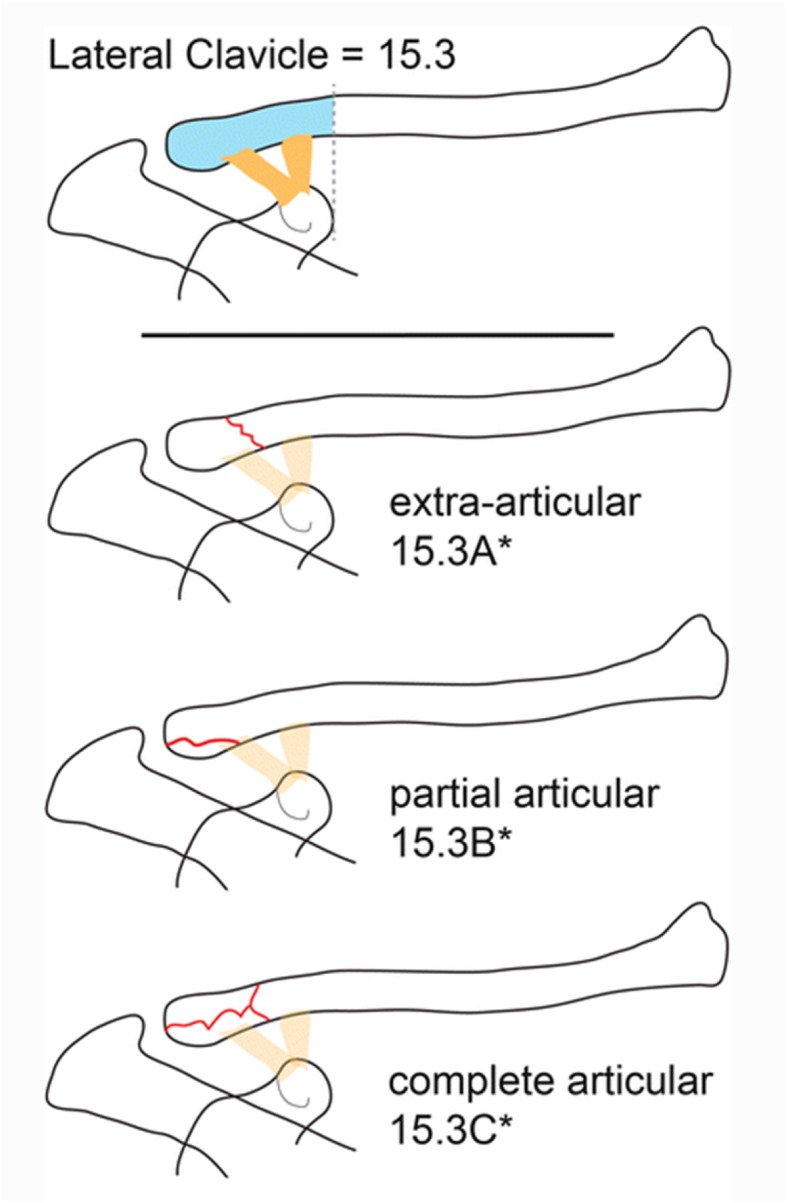
Summary of the OTA classification system [9] (adapted with permission from Sandstrom CK, Gross JA, Kennedy SA. Distal clavicle fracture radiography and treatment: a pictorial essay. Emergency radiology. 2018;25(3):311–9.) [17]

**Fig. 3:**
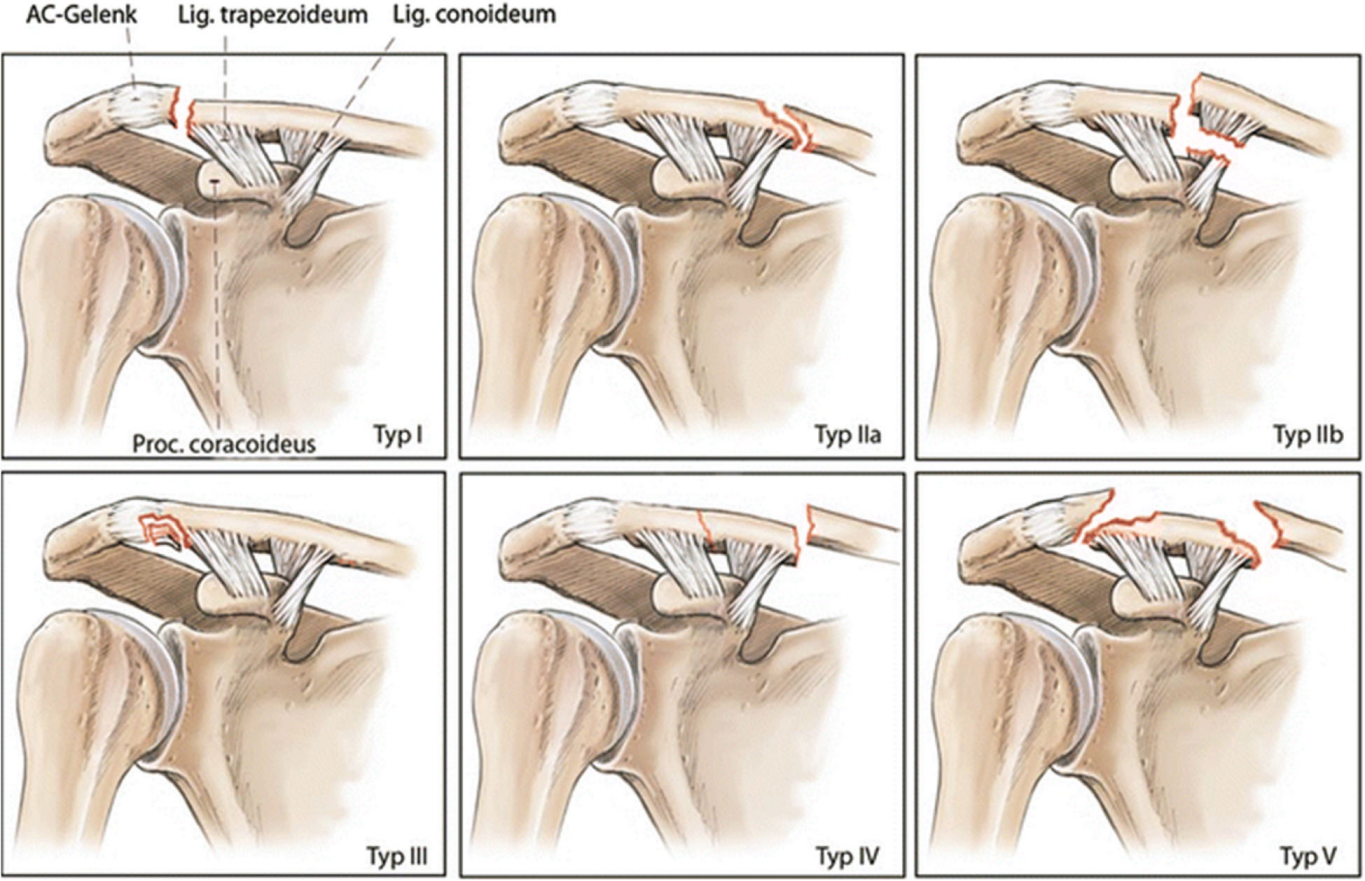
Summary of the Neer classification system [10] (adapted with permission from Lenich A, Imhoff AB. Fractures of the Clavicle. In: Doral MN, Karlsson J, editors. Sports Injuries: Prevention, Diagnosis, Treatment and Rehabilitation. Berlin, Heidelberg: Springer Berlin Heidelberg; 2015. p. 161–8.) [18]

**Fig. 4:**
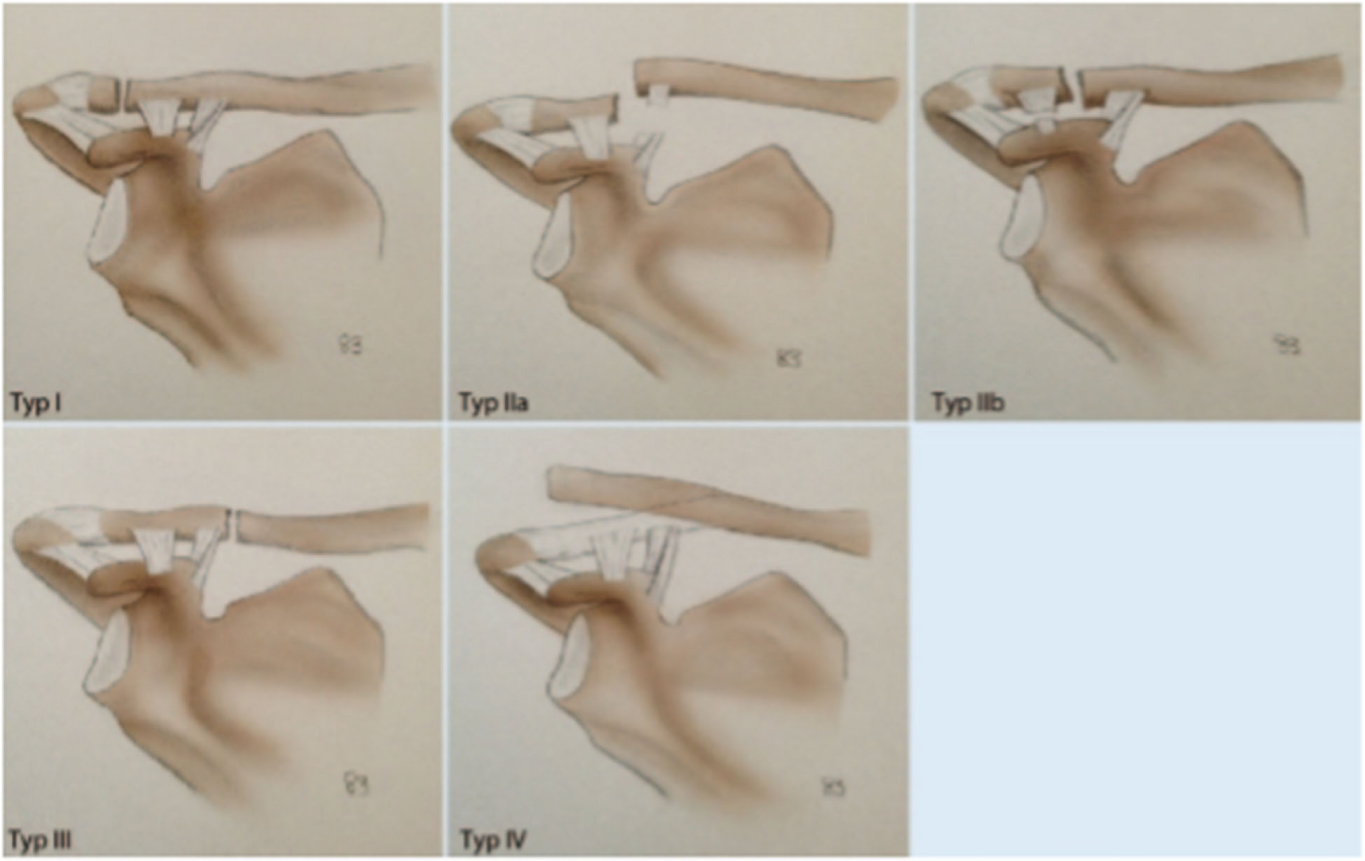
Summary of the Jäger/Breitner classification system [12] (adapted with permission from Schliemann B, Breiter S, Theisen C, Schneider KN, Kösters C, Raschke MJ, et al. Die laterale Klavikulafraktur – Grundlagen, OP-Indikationen, Versorgungstechniken. Obere Extremität. 2014;9(3):222–8.) [19]

A web-based survey was designed using the LimeSurvey Professional survey tool (Carsten Schmitz/LimeSurvey GmbH). X-ray images from 20 patients, without any patient identification signs were presented to the investigators. The X-ray images were available in random order and were classified independently by the investigators in consideration of the relationship between fracture pattern and the coracoclavicular ligaments (Fig. 1). For each case, the investigators were asked to classify the fracture according to the OTA, Neer, and Jäger/Breitner classification systems. To support the investigators in their understanding of the different classification systems, an original description and schematic illustrations of the OTA (Fig. 2), Neer (Fig. 3), and Jäger/Breitner (Fig. 4) classification systems were scanned and provided for each image. Investigators were blinded to all additional information including concomitant injuries, treatment modalities and outcomes. Time limits for completion of the survey were not imparted.

The online survey was performed at two different time points 3 months apart (first: 7th to 20th May 2018; second: 10th to 23rd September 2018). Between the two assessment intervals, no feedback was given to the investigators.

### Selection of radiographs

Inclusion criteria were: (1) adult patients (≥ 18 years), (2) the availability of a plain X-ray image of adequate adequality, and (3) acute fracture within less than 2 weeks after the initial trauma. Exclusion criteria were: (1) X-ray images of skeletally immature patients, (2) any other concomitant shoulder injury on the affected side, (3) any history of shoulder trauma or surgery on the affected side, (4) X-ray images of low quality, e.g. with artefacts or other technical defects, and (5) all dynamic imaging, e.g. CT and MRI.

The selected 20 X-ray images, including an anteroposterior and tangential views, were chosen by two experienced upper extremity specialized traumatologists with 7 and 14 years of post-residency experience. The selected X-ray images were considered to be representative of a wide range of adult lateral clavicle fracture patterns according to the OTA, the Neer, and the Jäger/Breitner classification systems with the attempt to match frequency in the subgroups of extraarticular, partial articular and complete articular fractures. Any patient identification signs were removed from the X-ray images. The X-ray images were scanned (300 dpi) and uploaded using a web-based survey system.

### Classification systems

#### OTA classification system

The OTA classification system [9] is a standardized method for describing fractures and dislocation. It uses an alphanumerical code based on injury location and type and serves as a mechanism to communicate data for clinical interaction and research [9]. Within this system, the clavicle is listed as no 15 and the lateral clavicle segment that begins at a perpendicular line to the medial edge of the coracoid process is encoded as 15.3. The coracoclavicular ligaments are part of this lateral segment. This classification system further subdivides into extraarticular (A), partial articular (B) and complete articular fractures (C) as well as three subgroups (a-c) depending on the relationship between fracture pattern and the coracoclavicular ligaments.

#### Neer classification system

The Neer classification system [10] is based on fracture location as well as location relative to and the integrity of the coracoclavicular ligaments [10, 11, 20]. Type I fractures are located lateral to the coracoclavicular ligaments with both conoid and trapezoid ligaments remaining intact. These fractures show minimal displacement and are considered stable. Type II fractures are located medial to the attachment of the coracoclavicular ligaments and are subdivided into groups A and B. In type IIA fractures occur medial to the intact remaining conoid and trapezoid ligaments, in type IIB fractures the conoid ligament is detached from the proximal fragment while the trapezoid ligament remains attached to the distal fragment. Both, type IIA and IIB fractures are unstable patterns associated with substantial medial clavicle displacement. In type III fractures, intra-articular extension into the acromioclavicular joint is present, while the conoid and trapezoid ligaments remain intact. These fractures, therefore, show only a minimal displacement and are considered stable. Type IV fractures occur in skeletally immature patients where a periosteal sleeve gets avulsed from the inferior cortex with intact remaining coracoclavicular ligaments, following lateral clavicle segment displacement superiorly through a tear in the thick periosteum. These fractures are supposed to be stable. Type V fractures show a comminuted fracture pattern with intact remaining conoid and trapezoid ligaments and significant medial clavicle displacement. These fractures are usually supposed to be unstable.

#### Jäger/Breitner classification system

Similar to the Neer classification system, the Jäger/Breitner classification system [12] is also based on the location of the fracture in relation to the coracoclavicular ligaments and their intactness. Type I fractures are located lateral to the coracoclavicular ligaments, while the conoid and trapezoid ligaments remain intact and without the involvement of the acromioclavicular joint. These fractures show minimal displacement and are supposed to be stable. Type II fractures are located at the level of the coracoclavicular ligaments. In type IIA fractures the medial conoid ligament is ruptured while the trapezoid ligament remains attached to the distal fragment. Type IIA fractures are unstable fractures with significant medial clavicle displacement. In type IIB fractures the medial conoid ligament remains intact while the trapezoid ligament is ruptured. Type IIB fractures are supposed to be stable fractures with minimal displacement. Type III fractures are located medial to the intact coracoclavicular ligament. Type IV fractures occur in skeletally immature patients where a periosteal sleeve gets avulsed from the inferior cortex with intact remaining coracoclavicular ligaments, following lateral clavicle segment displacement superiorly through a tear in the thick periosteum. These fractures are supposed to be stable.

### Statistical methods

The interobserver agreement was calculated to define the reliability between the investigator’s evaluation for each case. The intraobserver agreement was calculated based on the reliability of the individual investigators between the first and the second survey for each case.

To measure observer agreement for categorical data that occur above and beyond that related to chance alone, the kappa value and its variants are the currently most accepted methods [16, 21]. The kappa value is calculated as the difference of an observed agreement (P_O_) minus the chance agreement (P_C_) divided by the maximum possible agreement that is not related to chance (1- P_C_):

κ = (P_O_ - P_C_) / (1- P_C_) [16].

To calculate the observer agreement between more than two investigators we used in this study the Fleiss’ kappa value [22]. The achieved kappa values ranging from 0.0 (chance agreement) to 1.0 (complete agreement) [16]. To interpret the strength of agreement with the calculated kappa values of this study, the criteria for assessing the extent of agreement of Landis and Koch [16, 23] were used: κ > 0.80 (almost perfect); κ = 0.61 to 0.80 (substantial;) κ = 0.41 to 0.60 (moderate); κ = 0.21 to 0.40 (fair); κ = 0.00 to 0.20 (slight); κ < 0.00 (poor) (Table 1).

**Table 1:** Strength of agreement according to Kappa values

Kappa value	Strength of agreement
<0.00	Poor
0.00 to 0.20	Slight
0.21 to 0.40	Fair
0.41.0.60	Moderate
0.61 to 0.80	Substantial
0.81 to 1.00	Almost perfect

Interpretation of the strength of agreement with the calculated kappa values according to the criteria for assessing the extent of agreement of Landis and Koch

## Results

### Interobserver reliability

A total of 20 patients with a lateral clavicle fracture at a level 1 trauma center from 2014 to 2016 were included.

The overall interobserver reliability showed a fair agreement in all three classification systems (Table 2). The highest interobserver agreement with a mean kappa value of 0.338 showed the OTA classification system ranging from 0.350 in the group of the radiologists to 0.374 in the group of the trauma surgeons. The second best interobserver agreement with a mean kappa value of 0.330 showed the Jäger/Breitner classification system ranging from 0.306 in the group of the trauma surgeons to 0.382 in the group of the radiologists. The lowest interobserver agreement with a mean kappa value of 0.278 showed the Neer classification system ranging from 0.238 in the group of the trauma surgeons to 0.276 in the group of the radiologists.

**Table 2:** Kappa scores of the interobserver agreement

	OTA	Neer	Jäger and Breitner
Consultant trauma surgeons	0.374	0.238	0.306
Consultant radiologists	0.350	0.276	0.382
Overall	0.338	0.278	0.330

The overall interobserver reliability showed a fair agreement in all three classification systems

A better mean interobserver agreement was seen with the Trauma surgeon group when using the OTA classification system compared to the radiologist group. However, a better mean interobserver agreement was seen in the radiologist group while using the Neer and the Jäger/Breitner classification systems compared to the trauma surgeon group. Neither of the two specialist groups reached a better agreement level than fair.

### Intraobserver reliability

The overall intraobserver reliability was moderate for the OTA and the Jäger/Breitner classification systems, while the overall intraobserver reliability for the Neer classification system was fair. The highest intraobserver agreement with a mean kappa value of 0.461 was seen with the OTA classification system. The second best intraobserver agreement with a mean kappa value of 0.426 was seen with the Jäger/Breitner classification system. The lowest intraobserver agreement with a mean kappa value of 0.298 was seen with the Neer classification system. The kappa values of the intraobserver agreements showed in all classification systems a wide range with the OTA classification system ranging from 0.086 to 0.634, the Neer classification system ranging from 0.137 to 0.448, and a range from 0.154 to 0.625 of the Jäger/Breitner classification system (Table 3).

**Table 3:** Kappa scores of the intraoberserver agreement

Observers	OTA	Neer	Jäger and Breitner
A	0.568	0.153	0.492
B	0.446	0.405	0.300
C	0.567	0.241	0.186
D	0.498	0.279	0.470
E	0.446	0.342	0.625
F	0.558	0.228	0.380
G	0.086	0.379	0.583
H	0.373	0.448	0.489
I	0.634	0.137	0.307
K	0.429	0.404	0.570
Mean	0.461	0.302	0.440

The kappa values of the intraobserver agreements showed in all classification systems a wide range

For both, the inter- and the intraobserver reliability, the OTA classification system showed the highest mean kappa values, followed by the Jäger/Breitner classification system. The lowest mean kappa values for the interobserver and the intraobserver reliability was seen with the Neer classification system.

## Discussion

In the United States, the incremental cost of clavicle fractures in patients of 65 to 69 years of age was $12,682 and in patients of 70 to 74 years of age was $12,744 [24]. Due to the complexity of lateral clavicle fractures, a variety of different treatment options are described and remain a topic of controversy [1–8]. The relationship between fracture pattern and coracoclavicular ligament integrity impacts lateral clavicle stability and substantially influences treatment. The non-union rate of 22 to 50% for unstable lateral clavicle fractures treated non-operatively supports the consideration of operative treatment [10, 20, 25–28]. This emphasizes the importance of a reliable and valid classification system that distinguishes whether or not a lateral clavicle fracture is stable. Only a few of the classification systems for lateral clavicle fractures, e.g. Neer, modified Neer classification system, and the new classification system for lateral clavicle fractures by Cho et al. have been evaluated for their reliability [13–15].

The aim of this study, therefore, was to verify the reliability of three commonly used lateral clavicle fracture classification systems (OTA, Neer, Jäger/Breitner) [9–12], by evaluating the interobserver and the intraobserver agreement amongst two specialist groups. To our knowledge, this is the first study that evaluates the reliability of lateral clavicle fracture classification systems and contrasts different specialist groups.

In general, a broad variety of classification systems is commonly used by trauma and orthopedic surgeons to initiate appropriate treatment. Only a few of these classification systems have been tested for their reliability [16]. Garbuz et al. pointed out that inter- and intraobserver agreement among medical classification systems showed poor reliability [16]. Furthermore, they question whether newer systems would fare any better [16]. Nevertheless, despite their limitations, fracture classification systems are important to categorize the management problems and guide the physicians’ treatment algorithms [16].

In this study, the overall interobserver reliability showed a fair agreement in all three classification systems, ranging from 0.338 (OTA classification system) and 0.330 (Jäger/Breitner classification system) to 0.278 (Neer classification system). These results were similar to those reported for other fracture classification systems [16] such as those for distal radius fractures [29].

Bishop et al. [14] demonstrated fair interobserver agreement among 22 shoulder/sports medicine fellowship-trained orthopedic surgeons when using the Neer classification system for lateral clavicle fractures. Cho et al. [13] also rated the inter- and intraobserver agreement among nine shoulder specialists and nine orthopedic fellows as fair when using the modified Neer classification system. These results were corroborated by the present study, where both specialist groups reached a no better agreement than fair in all three evaluated classification systems. Regarding the interobserver reliability of the present study, the group of the trauma surgeons showed a better mean interobserver agreement than the group of the radiologist applying the OTA classification system, while using the Neer and the Jäger/Breitner classification systems the group of the radiologist had a better mean interobserver agreement. This may be owed to the preference and the more frequent use of the OTA classification system in the daily business in the group of trauma surgeons. While all investigators in this study were independent and experienced consultants, either in the field of musculoskeletal radiology or in the field of trauma surgery, these results are concordant with the results of other reliability evaluating studies [14], that emphasized the importance of assignment of experts to test the classification system itself [30]. Furthermore, it underscores the need for both a meticulous clinical examination as well as imaging in determining individualized treatment options. As Bishop et al. [14] rated the fracture stability and size of the distal fragment as important factors in making the decision to operate and which implant to use, Cho et al. [15] devised a new classification system for lateral clavicle fractures by taking into account fracture displacement and stability as well as fracture location. This new classification system, which is not a widespread concept in practice and therefore not tested in this study, demonstrated moderate interobserver (κ = 0.434) and substantial intraobserver (κ = 0.644) reliability [15]. In their study, Cho et al. included the evaluation of a total of eight investigators, four shoulder specialists and four orthopedic fellows at two different time points with only 4 weeks between the two evaluations. This short interval between the two evaluations is a point of criticism of this study and could be an explanation of their documented substantial intraobserver reliability.

By choosing an appropriate time window of 3 months between the two evaluations, the overall intraobserver reliability in the present study was moderate for the OTA and the Jäger/Breitner classification systems, while the overall intraobserver reliability for the Neer classification system was fair.

Similar to previous evaluations of other orthopedic classification systems [16, 29, 31–34] the kappa values of the intraobserver agreements of this study showed in all classification systems a wide variability.

The wide variability of the kappa values and the fair to at best moderate reliability of the three classification systems that we evaluated is probably due to both the relationship of the fracture to the coracoclavicular ligaments and the inherent complexity of each classification system. In order to better assess the fracture and its relation to the coracoclavicular ligaments, a CT may be performed. But this does not reflect the usual diagnostic workup of most of the emergency wards. Furthermore, Cho et al. [13] demonstrated in their study in 2015, that additional 3D CT did not improve the overall level of interobserver or intraobserver agreement over the modified Neer classification system.

## Conclusions

The low agreement results for lateral clavicle fracture classification systems, shown in the data from this study, demonstrated limited reliability which calls their validity into question. We should recognize there is considerable inconsistency in how physicians classify lateral clavicle fractures and therefore any conclusions based on fracture classification should be recognized as being somewhat subjective.

## Data Availability

The raw data used in the analyses of this study are available in the authors’ database.
